# Plant-Based Diets and Cardiometabolic Diseases in a Cohort of Iranian Adults: A Systematic Review of Evidence from the Tehran Lipid and Glucose Study

**DOI:** 10.5812/ijem-167232

**Published:** 2025-10-31

**Authors:** Nazanin Moslehi, Mahdieh Golzarand, Parvin Mirmiran, Fereidoun Azizi

**Affiliations:** 1 Nutrition and Endocrine Research Center, Research Institute for Endocrine Disorders, Research Institute for Endocrine Sciences, Shahid Beheshti University of Medical Sciences, Tehran, Iran; 2 Department of Clinical Nutrition and Dietetics, Faculty of Nutrition Sciences and Food Technology, National Nutrition and Food Technology Research Institute, Shahid Beheshti University of Medical Sciences, Tehran, Iran; 3 Endocrine Research Center, Research Institute for Endocrine Disorders, Research Institute for Endocrine Sciences, Shahid Beheshti University of Medical Sciences, Tehran, Iran

**Keywords:** Dietary Approaches to Stop Hypertension, Mediterranean Diet, Cardiometabolic Risk Factors, Cardiovascular Diseases, Diabetes Mellitus

## Abstract

**Background:**

Although numerous studies have demonstrated that plant-based diets can reduce the risk of cardiometabolic diseases, their applicability and health benefits within Iran remain unconfirmed.

**Objectives:**

This systematic review aimed to clarify the findings from the Tehran Lipid and Glucose Study (TLGS) regarding the benefits of these recommended diets against cardiometabolic diseases in the Iranian population.

**Methods:**

A systematic literature search was conducted in PubMed/Medline, Web of Science, and Scopus until September 2025. Studies that assessed the association between plant-based diets, including the Mediterranean diet (MeDi), dietary approaches to stop hypertension (DASH), Mediterranean-DASH intervention for neurodegenerative delay (MIND), Nordic, and Portfolio diets, and cardiometabolic diseases [i.e., cardiovascular disease (CVD) and type 2 diabetes mellitus (T2DM)] or their risk factors [i.e., overweight/obesity, hyperglycemia or insulin resistance (IR), dyslipidemia, and hypertension (HTN)] within the TLGS framework were included.

**Results:**

From the 1,293 articles, 13 studies were included. Nine studies were identified on the MeDi score and cardiometabolic diseases; most found no significant associations. Five studies were conducted on the DASH score, which revealed an inverse association with risk of IR but no other outcomes. Two studies assessed the link between the MIND and Nordic diets and CVD with protective associations. There was one study on the Portfolio diet that showed no association with T2DM risk.

**Conclusions:**

Within the TLGS, the MeDi and DASH diet scores have been studied more than other plant-based diets, yet most findings show no beneficial association with cardiometabolic diseases. Conversely, recent findings regarding the MIND and Nordic diets indicate a significant inverse association with CVD incidence. Given the scarcity of research in this area, further investigation into plant-based diets and cardiometabolic health within the TLGS is warranted.

## 1. Introduction

Cardiovascular diseases (CVDs) and type 2 diabetes mellitus (T2DM) have represented significant public health challenges for decades due to their rising global prevalence and substantial contribution to mortality worldwide (1-3). In Iran, CVDs are the leading cause of mortality and disability, responsible for approximately 46% of all deaths and a significant proportion of the nation’s disease burden, driven in part by T2DM (4, 5). Risk factors such as adiposity, insulin resistance (IR), dyslipidemia, and high blood pressure (BP) increase the likelihood of developing CVDs and T2DM (6-9). In this context, dietary patterns have emerged as a cornerstone for both the prevention and management of cardiometabolic diseases (10, 11). Among these, plant-based diets have been the subject of growing research interest.

Plant-based diets are dietary patterns characterized by a high consumption of plant food groups, including fruits, vegetables, whole grains, legumes, nuts, and seeds, with restricted intake of animal products (12). This term encompasses a spectrum of dietary practices, ranging from vegetarian and vegan diets that eliminate animal products to semi-vegetarian patterns that limit them. The Mediterranean diet (MeDi), dietary approaches to stop hypertension (DASH) diet, Mediterranean-DASH intervention for neurodegenerative delay (MIND) diet, Nordic diet, Portfolio diet, and Paleolithic diet, as well as the Plant-Based Diet Index (PDI) and the planetary health diet, are all examples of these types of diets (12).

The Tehran Lipid and Glucose Study (TLGS) is a population-based prospective cohort study initiated in 1999. It was designed to investigate the prevalence and risk factors for non-communicable diseases within an urban Iranian population (13). The TLGS provides a unique and invaluable dataset to explore the relationship between diet and health in a West Asia context, where dietary habits and disease patterns may differ from Western populations (14, 15). Over multiple follow-up examinations, detailed nutritional, clinical, and biochemical data have been collected in the TLGS, allowing for a robust investigation of how long-term dietary exposures influence health outcomes (16).

Although numerous studies have demonstrated that plant-based diets can reduce the risk of cardiometabolic diseases (17-19), their applicability and health benefits within Iran’s distinct socio-cultural context remain unconfirmed. A systematic review focusing specifically on the findings from the TLGS would provide crucial, population-specific evidence. It would clarify whether these internationally recommended diets confer protective benefits against cardiometabolic diseases in Tehran’s population and identify which patterns are most strongly associated with positive outcomes.

## 2. Objectives

Consequently, this systematic review aims to consolidate the existing evidence from the TLGS regarding the associations between adherence to plant-based diets and two particular cardiometabolic diseases (CVDs and T2DM), along with their principal risk factors (adiposity, IR or dysglycemia, dyslipidemia, and high BP).

## 3. Materials and Methods

### 3.1. Protocol and Registration

A protocol for this systematic review was not prospectively registered. This is because the study was limited to published articles from the TLGS and was not intended to be a comprehensive systematic review of all available sources on the topic. However, the review process was planned and conducted in full compliance with the Preferred Reporting Items for Systematic Reviews and Meta-Analyses (PRISMA) 2020 checklist.

### 3.2. Search Strategy

We comprehensively searched three databases — PubMed/Medline, Web of Science, and Scopus — from inception until September 2025 using the following key words: Cerebrovascular disorders, CVDs, coronary heart disease (CHD), heart disease, adverse cardiac event, cardiovascular death, cardiovascular mortality, stroke, diabetes mellitus, cardiometabolic, IR, glucose intolerant, hyperglycemia, impaired glucose tolerance, impaired fasting glucose, dyslipidemia, hypertension (HTN), hyperlipidemia, obesity, plant-based diet, plant-based nutrition, DASH, vegetarian, vegan, dietary pattern, MeDi, Nordic diet, MIND diet, Palaeolithic diet, PDI, portfolio diet, and TLGS. The search strategy is presented in Table 1. 

**Table 1.** The Search Strategy

Electronic Databases	Search Strategy	Records
**PubMed/Medline**	((“Cerebrovascular Disorders”[Mesh] OR “Cardiovascular Diseases”[Mesh] OR “cerebrovascular dis*”[Title/Abstract] OR “cardiovascular diseases”[Title/Abstract] OR “CVD”[Title/Abstract] OR “coronary heart disease”[Title/Abstract] OR “CHD”[Title/Abstract] OR “heart disease”[Title/Abstract] OR “myocardial infarction”[Title/Abstract] OR “MI”[Title/Abstract] OR “Adverse Cardiac Event*”[Title/Abstract] OR “cardiovascular death”[Title/Abstract] OR “cardiovascular mortality”[Title/Abstract] OR “stroke”[Mesh] OR “Stroke*”[Title/Abstract] OR "Diabetes Mellitus"[Mesh] OR "cardiometabolic*"[Title/Abstract] OR diabet*[tiab] OR "insulin resistan*"[tiab] OR "glucose intoleran*"[tiab] OR "hyperglycemi*"[tiab] OR "hyperglycemi*"[Mesh] OR “prediab*”[Title/Abstract] OR "impaired glucose tolerance"[tiab] OR "impaired fasting glucose"[tiab] OR dyslipidemia[tiab] OR dyslipidemia[Mesh] OR hypertension[tiab] OR hypertension[Mesh] OR hyperlipidemia[Mesh] OR “Obesity”[Mesh] OR “overweight”[Mesh]) AND ("Diet, Plant-Based" [Mesh] OR "Diet, Vegetarian"[Mesh] OR "Diet, Plant-Based"[Title/Abstract] OR "Plant-Based Diet*"[Title/Abstract] OR "Plant Based Diet"[Title/Abstract] OR "Plant-Based Nutrition"[Title/Abstract] OR "Plant Based Nutrition"[Title/Abstract] OR "vegetarian"[Title/Abstract] OR vegan*[Title/Abstract] OR veget*[Title/Abstract] OR plant*[Title/Abstract] OR "vegetable"[Title/Abstract] OR "dietary pattern"[Title/Abstract] OR "diet, Mediterranean"[MeSH] OR "Mediterranean"[tiab] OR "Dietary Approaches To Stop Hypertension"[Mesh] OR "Dietary Approaches To Stop Hypertension"[tiab] OR DASH[tiab] OR "Nordic diet"[Title/Abstract] OR "MIND diet"[Title/Abstract] OR "Paleolithic diet"[Title/Abstract] OR "planetary health diet index"[Title/Abstract] OR "Mediterranean-DASH Intervention for Neurodegenerative Delay"[tiab] OR portfolio diet[tiab]) AND ("Tehran lipid and glucose study"[tw] OR "TLGS"[tw]))	73
**Web of Science**	TS=(“Cerebrovascular Disorders” OR “Cardiovascular Diseases” OR “CVD” OR “coronary heart disease” OR “CHD” OR “ischemic heart disease” OR “myocardial infarction” OR “MI” OR “Adverse Cardiac Event*” OR “cardiovascular death” OR “cardiovascular mortality” OR “Stroke*” OR "Diabetes Mellitus" OR "cardiometabolic*" OR diabet* OR "insulin resistan*" OR "glucose intoleran*" OR "hyperglycemi*" OR "hyperglycemi*" OR “prediab*” OR "impaired glucose tolerance" OR "impaired fasting glucose" OR dyslipidemia OR dyslipidemia OR hypertension OR hypertension OR hyperlipidemia OR obesity OR overweight) AND TS=("Diet, Plant-Based" OR "Plant-Based Diet*" OR "Plant Based Diet" OR "Plant-Based Nutrition" OR "Plant Based Nutrition" OR "vegetarian" OR vegan* OR veget* OR plant* OR "vegetable" OR "dietary pattern" OR "Mediterranean" OR "Dietary Approaches To Stop Hypertension" OR DASH OR "Nordic diet" OR "MIND diet" OR "Paleolithic diet" OR "planetary health diet index" OR "Mediterranean-DASH Intervention for Neurodegenerative Delay" OR "portfolio diet") AND TS=("Tehran lipid and glucose study" OR "TLGS")	102
**Scopus**	TITLE-ABS-KEY(“Cerebrovascular Disorders” OR “Cardiovascular Diseases” OR “CVD” OR “coronary heart disease” OR “CHD” OR “ischemic heart disease” OR “myocardial infarction” OR “MI” OR “Adverse Cardiac Event*” OR “cardiovascular death” OR “cardiovascular mortality” OR “Stroke*” OR "Diabetes Mellitus" OR "cardiometabolic*" OR diabet* OR "insulin resistan*" OR "glucose intoleran*" OR "hyperglycemi*" OR "hyperglycemi*" OR “prediab*” OR "impaired glucose tolerance" OR "impaired fasting glucose" OR dyslipidemia OR dyslipidemia OR hypertension OR hypertension OR hyperlipidemia OR obesity OR overweight) AND TITLE-ABS-KEY("Diet, Plant-Based" OR "Plant-Based Diet*" OR "Plant Based Diet" OR "Plant-Based Nutrition" OR "Plant Based Nutrition" OR "vegetarian" OR vegan* OR veget* OR plant* OR "vegetable" OR "dietary pattern" OR "Mediterranean" OR "Dietary Approaches To Stop Hypertension" OR DASH OR "Nordic diet" OR "MIND diet" OR "Paleolithic diet" OR "planetary health diet index" OR "Mediterranean-DASH Intervention for Neurodegenerative Delay" OR "portfolio diet") AND ALL("Tehran lipid and glucose study" OR "TLGS")	1118

### 3.3. Screening and Data Extraction

The initial search results were exported to EndNote X7 to identify relevant studies. After removing the duplicate studies, two independent researchers (N.M. and M.G.) screened eligible papers based on their titles and abstracts and subsequently assessed them based on full-text content. Our inclusion criteria were as follows: (1) Be observational in design and conducted within the TLGS; (2) include adult participants (≥ 18 years); (3) define exposure as a plant-based dietary pattern (specifically, the MeDi, DASH, MIND, Portfolio, Nordic diet, Palaeolithic diet, planetary health diet, and plant-based diet indices) (12); and (4) report cardiometabolic diseases (i.e., CVD or T2DM) or their risk factors (adiposity, IR or dysglycemia, dyslipidemia, and high BP) as outcomes. We excluded randomized controlled trials, those that included children or adolescents, focused on other dietary patterns or scores, assessed non-cardiometabolic disease, or examined outcomes of interest continuously.

The following information was extracted from studies: First author, publication year, study design, specific plant-based diet examined, sample size, participant age, follow-up duration, measured outcomes, and covariates adjusted for in the analysis.

Given that all studies included in this systematic review are secondary analyses of the single, well-defined TLGS cohort, a formal quality assessment was not performed. The core methodological aspects related to participant selection, baseline data collection, and follow-up procedures are uniform across all publications, as they are inherent to the design and execution of the TLGS itself.

## 4. Results

### 4.1. Study Selection

**Figure 1.**
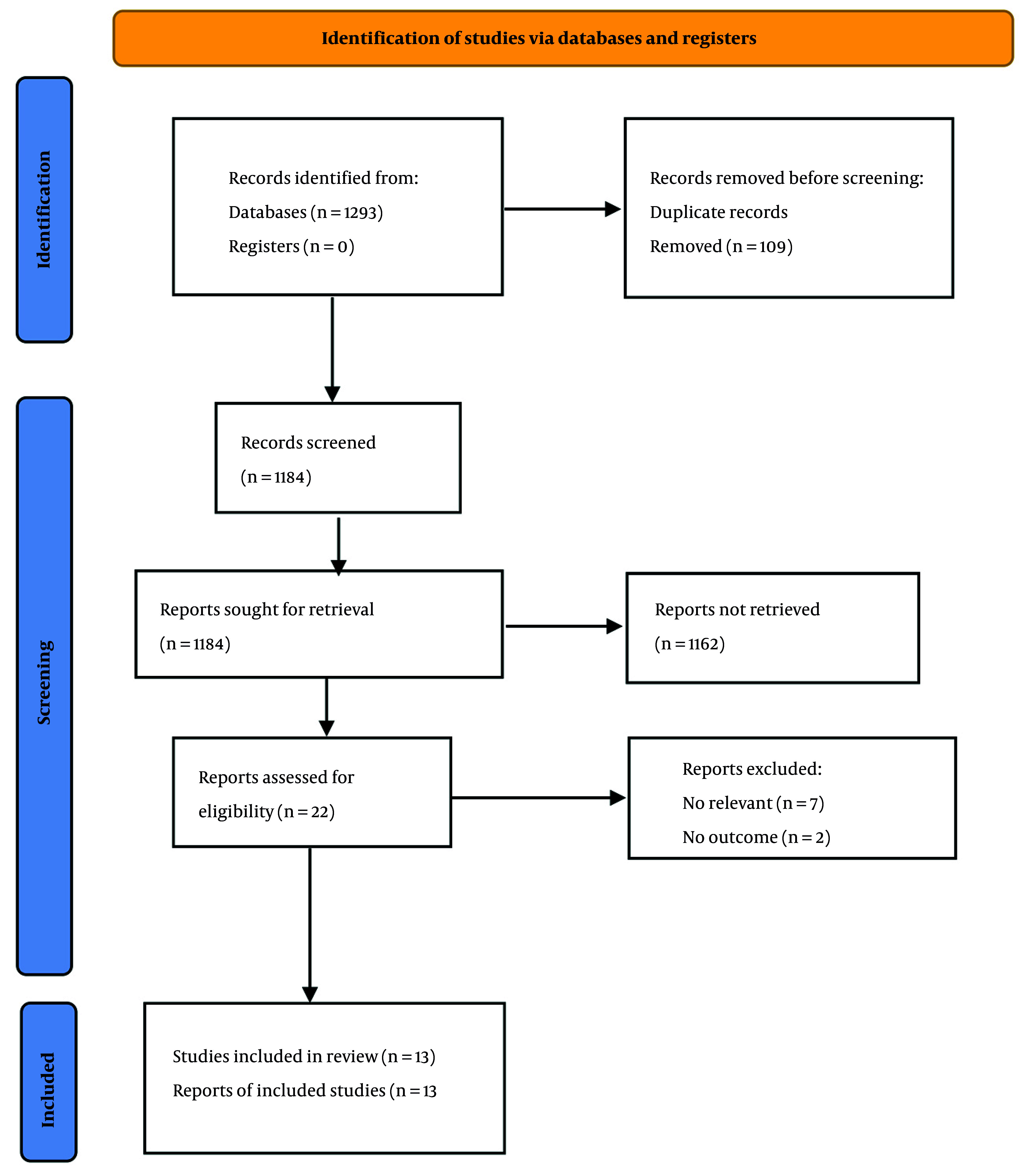
Flowchart of included studies for systematic review

Figure 1 shows the flowchart of the included studies. We identified 1,293 eligible papers by our initial search in electronic databases, including PubMed/Medline (n = 73), Scopus (n = 1,118), and Web of Science (n = 102). One hundred nine papers were eliminated due to duplicate publication, and 1,184 papers were assessed. We excluded 1,162 papers based on their titles and abstracts, and 22 papers were evaluated for further information. Among them, seven irrelevant papers were excluded (20-26), and two were removed due to a lack of outcomes of interest (27, 28). Finally, 13 papers were included in the present systematic review (29-41).

### 4.2. Characteristics of Studies

Table 2 presents the characteristics of the included studies. Five studies addressed the MeDi score (29, 31-33, 40), one study examined the DASH score (34), and three studies assessed both MeDi and DASH scores (30, 35, 39). Additionally, one study compared the MeDi, DASH, and MIND scores (37). A single independent study assessed the MIND score (36), Nordic score (38), and Portfolio diet (41). No studies were identified that examined the Palaeolithic diet, planetary health diet, or plant-based diet indices. Three studies assessed CVD risk (36, 38, 40), five studies examined T2DM (32, 33, 35, 39, 41), and five studies evaluated cardiometabolic risk factors (29, 31, 34, 37). The sample sizes of the studies ranged from 334 to 6,112.

**Table 2.** Summary of Studies Investigating the Association Between the Plant-Based Diets and Cardiometabolic Outcomes in the Tehran Lipid and Glucose Study

Author, y (Ref.)	Study Design	Dietary Exposure	Subjects	Duration (y)	Outcome Definitions	Adjustment	Results for Highest vs. Lowest Groups
**Mirmiran et al., 2015 (** 29 **)**	Prospective	MeDi	2241 (18 - 74 y)	3	Abdominal obesity (WC ≥ 90 cm); High FSG (FSG ≥ 100 mg/dL or anti-diabetic medications); High TGs (≥150 mg/dL or lipid-lowering medications; Low HDL-C (< 40 mg/dL in men or < 50 mg/dL in women or lipid-lowering medications); High BP (SBP/DBP ≥ 130/85 or antihypertensive medications	Age, sex, smoking, physical activity, energy intake, BMI, and BMI changes	OR T3 vs. T1 for high WC: 0.74 (0.48, 1.13) ^a^; OR T3 vs. T1 for high FSG:1.01 (0.73, 1.39); OR T3 vs. T1 for high TGs: 0.81 (0.48, 1.40); OR T3 vs. T1 for low HDL-C: 0.82 (0.48, 1.40); OR T3 vs. T1 for high BP: 0.89 (0.64, 1.22)
**Esfandiari et al., 2017 (** 30 **)**	Prospective	MeDi, DASH	927	3	IR (HOMA-IR ≥ 3.2)	Age, sex, BMI, WC, smoking, physical activity, anti-diabetic medications, serum insulin, HOMA-IR, energy intake	OR T3 vs. T1 for MeDi: 1.07 (0.60, 1.91); OR T3 vs. T1 for DASH: 0.39 (0.20,0.76)
**Hosseini-Esfahani et al., 2017 (** 31 **)**	Nested case-control study	MeDi	1254 (> 18 y)	-	Obesity (BMI ≥ 30 kg/m^2^); Abdominal obesity (WC ≥ 95 cm); High WHR (WHR ≥ 0.8 in men and ≥ 0.9 in women)	Age, sex, education, smoking, physical activity, and energy intake	OR Q4 vs. Q1 for obesity: 0.67 (0.34, 1.29) ^b^; OR Q4 vs. Q1 for abdominal obesity: 1.37 (0.75, 2.52); OR Q4 vs. Q1 for high WHR: 0.77 (0.35, 1.41)
**Khalili-Moghadam et al., 2018 (** 32 **)**	Prospective	MeDi	2139 (20 - 70 y)	6	T2DM (FSG ≥ 126 mg/dL or 2-h SG ≥ 200 mg/dL, or anti-diabetic medications)	Diabetes risk score	HR T3 vs. T1: 0.48 (0.27, 0.83)
**Ramezan et al., 2019 (** 33 **)**	Nested case-control	MeDi	561 (> 20 y)	-	T2DM (FSG ≥ 126 mg/dL or 2-h SG ≥ 200 mg/dL, or anti-diabetic medications)	Matched on age, sex, and date of data collection and controlled for family history of diabetes, WC, BMI, education, smoking, physical activity, energy intake, hypercholesterolaemia, and hypertension	OR high vs. low score: 0.93 (0.44,1.96)
**Ramezankhani et al., 2021 (** 34 **)**	Prospective	DASH	4793 (≥ 18 y)	6.3	HTN (SBP ≥ 140 mmHg or DBP ≥ 90 mmHg or taking antihypertensive medications)	Age, sex, BMI, smoking, diabetes status, physical activity, TGs, family history of CVD, and salt and energy intakes	HR Q4 vs.Q1: 1.19 (0.95, 1.49)
**Esfandiar et al., 2022 (** 35 **)**	Prospective	MeDi, DASH	6112 (≥ 18 y)	6.6	T2DM (FSG ≥ 126 mg/dL or 2-h SG ≥ 200 mg/dL, or antidiabetic medications)	Age, sex, diabetes risk score, physical activity, smoking, dietary fiber, and energy intake	HR Q4 vs. Q1 for MeDi:1.06 (0.87,1.30); HR Q4 vs. Q1 for DASH: 1.13 (0.88,1.46)
**Golzarand et al., 2022 (** 36 **)**	Prospective	MIND	2863 (≥ 19 y)	10.6	CVD events (CHD, stroke, and CVD-related mortality)	Sex, age, BMI, smoking, SES, energy intake, diabetes, and hypertension	HR T3 vs. T1: 0.68 (0.47, 0.97)
**Razmpoosh et al., 2022 (** 37 **)**	Prospective	MeDi, DASH, MIND	2706 (≥ 18 y)	7.4	HTN (SBP ≥ 140 mmHg or DBP ≥ 90 mmHg or taking antihypertensive medications)	Age, sex, physical activity, education, premature family history of CVD, smoking, BMI, prevalent of diseases, aspirin, and energy, caffeine and olive intakes	HR Q4 vs. Q1 for MeDi: 0.76 (0.53, 1.09); HR Q4 vs. Q1 for DASH: 0.99 (0.79, 1.25); HR Q4 vs. Q1 for MIND: 1.08 (0.86, 1.36)
**Mirmiran et al., 2023 (** 38 **)**	Prospective	Nordic	2918	10.6	CVD events (CHD, stroke, and CVD-related mortality)	Sex, age, BMI, smoking, dyslipidemia, diabetes, hypertension, changes in weight, and energy intake	HR Q4 vs. Q1: 0.40 (0.27, 0.60)
**Mirmiran et al., 2023 (** 39 **)**	Prospective	MeDi, DASH	334 (≥ 21 y)	9	Pre-DM progression to T2DM; Pre-DM regression to NG	Age, sex, diabetes risk score, weight changes, smoking, and physical activity	OR T2DM per one-score of MeDi: 1.06 (0.86, 1.29); OR T2DM per one-score of DASH: 1.03 (0.96, 1.11); OR NG per one-score of MeDi: 1.02 (0.86, 1.29); OR NG per one-score of DASH: 0.98 (0.92, 1.06)
**Norouzzadeh et al., 2024 (** 40 **)**	Prospective	MeDi-DQI	955 (smokers ≥ 30 y)	8	CVD events (CHD, stroke, and CVD-related mortality); All cause mortality	Age, SBP, FSG, occupation, BMI, physical activity, energy intake, marital status, and education	HR T3 vs. T1 for CVDs: 0.62 (0.26, 1.05); HR T3 vs. T1 for all-cause mortality: 3.45 (1.12, 10.6)
**Malmir et al., 2025 (** 41 **)**	Prospective	Potfolio	2,188 (≥ 21 y)	8.9	T2DM (FSG ≥ 126 mg/dL or 2-h SG ≥ 200 mg/dL, or anti-diabetic medications)	Age, sex, BMI, energy, physical activity, and diabetic risk score	HR T3 vs. T1: 0.91 (0.63, 1.30)

Abbreviations: BMI, Body Mass Index; BP, blood pressure; CHD, coronary heart disease; CVDs, cardiovascular diseases; DASH, dietary approaches to stop hypertension; DBP, diastolic blood pressure; FSG, fasting serum glucose; HDL-C, high-density lipoprotein cholesterol; HOMA-IR, homeostatic model assessment for insulin resistance; 2-h SG, 2-hour serum glucose; HR, hazard ratio; HTN, hypertension; IR, insulin resistance; MeDi, Mediterranean diet; MeDi-DQI, Mediterranean Dietary Quality Index; MIND, Mediterranean-DASH intervention for neurodegenerative delay; OR, odds ratio; SBP, systolic blood pressure; T2DM, type 2 diabetes; TGs, triglycerides; WC, waist circumference; WHR, waist-to-hip ratio.

^a^ Results were reported for the Trichopoulou et al. (42) MeDi score.

^b^ Results were reported only for a subgroup with more genetic risk alleles of FTO variants (genetic risk score ≥ 6).

### 4.3. Mediterranean Diet Score

Research from the TLGS has examined the association between adherence to the MeDi score and various cardiometabolic parameters, including abdominal and general obesity, elevated fasting serum glucose (FSG), IR, T2DM, HTN, high triglycerides (TGs), low high-density lipoprotein cholesterol (HDL-C) in the general population, and CVDs and all-cause mortality in smokers (29-33, 35, 37, 39, 40).

The MeDi score adherence was primarily assessed using the Trichopoulou et al. (42) method. One study (29) supplemented this approach by incorporating sex-specific absolute cut-off values proposed by Sofi et al. (43). Due to unavailable alcohol intake data, an 8-component version of the Trichopoulou Index was used, yielding an attainable score of 0 - 8, while the adapted Sofi et al. index ranged from 0 - 14. One prospective study utilized the Mediterranean-Dietary Quality Index (MeDi.DQI), which evaluates seven dietary components with a total score range of 0 - 14 (40). It is important to note that, contrary to other indices, a higher MeDi.DQI score signifies poorer dietary adherence.

An initial investigation of 2,241 adults found no significant differences in the odds of high waist circumference (WC), elevated fasting glucose, high TGs, low HDL-C, or HTN across MeDi score tertiles after a three-year follow-up (29). Similarly, a nested case-control study examining MeDi score in the context of fat mass and obesity-associated (FTO) genetic variants reported a lower, but non-significant, probability of obesity across MeDi score quartiles in individuals with a higher genetic risk (31). A prospective study of 2,706 adults also showed no significant association between MeDi score and HTN incidence over 7.4 years (37).

Regarding glucose metabolism, a three-year prospective study of 927 adults found no significant association between MeDi score tertiles and IR (30). However, the association between the MeDi score and T2DM was inconsistent across studies (32, 33, 35). One prospective study (35) and one nested case-control study (33) found no significant link to T2DM, but another prospective study with a six-year follow-up period found a significant inverse association (32). In the latter study, the risk of developing T2DM was significantly lower in the second [hazard ratio (HR): 0.64; 95% CI: 0.43 - 0.94] and third (HR: 0.48; 95% CI: 0.27 - 0.83) MeDi tertiles relative to the first tertile (32). Finally, an eight-year prospective study of 955 smokers found no significant association between the MeDi.DQI score and CVDs (40).

### 4.4. Dietary Approaches to Stop Hypertension Diet Score

A total of five cohort studies (30, 34, 35, 37, 39) have assessed the association between the DASH score and outcome of interest. Four studies (30, 34, 35, 39) used the Fung et al. (44) approach, and one study (37) applied the Epstein et al. (45) method to calculate the DASH score. A prospective study involving 927 adults with a mean age of 40.3 years examined the association between the DASH score and the incidence of IR. They reported an inverse association between the highest tertile of DASH score and odds of IR over the 3-year follow-up [odds ratio (OR): 0.39; 95% CI: 0.20 - 0.76] (30). In another cohort, 4,793 adults (mean age 40.3 years), no significant relationship between a higher adherence to the DASH score and the risk of HTN was established in the fully adjusted model (HR: 1.19; 95% CI: 0.95 - 1.49) after 6.3 years of follow-up (34). Similar results were identified in the other cohort, including 2,706 adults (mean age 37.9 years) over 7.4 years, indicating no link between the highest quartile of the DASH score and the risk of HTN (HR: 0.99; 95% CI: 0.79 - 1.25) (37). In a further study of 6,112 adults with normoglycemia, after adjustment for all potential confounders, risk of T2DM incidence was not significant across quartiles of the DASH score (HR: 1.13; 95% CI: 0.88 - 1.46) (35). In a separate cohort, in individuals with pre-diabetes, a higher adherence to the DASH score was not correlated to the progression to normal glycemia (OR: 0.98; 95% CI: 0.92 - 1.06) or T2DM (OR: 1.03; 95% CI: 0.96 - 1.11) (39).

### 4.5. Mediterranean-Dietary Approaches to Stop Hypertension Intervention for Neurodegenerative Delay Score

Two studies assessed the association between the MIND diet and cardiometabolic diseases (36) and their risk factors (37). Both studies had estimated the MIND score applying the Morris et al. approach (46). This approach computed the MIND score based on consumption of 15 foods, including whole grains, poultry, fish, green leafy vegetables, other vegetables, nuts, beans, olive oil, berries, wine, butter and margarine, cheese, red meat and goods, fast fried foods, and pastries and sweets, that influence brain health.

Results of a cohort study on 2,863 adults free from cardiovascular events revealed an inverse association between a 10.6-year CVD risk in individuals who were in the highest tertile of the MIND score compared with those in the lowest tertile (HR: 0.68; 95% CI: 0.47 - 0.97). Each score increase in the MIND score was related to a 16% lower risk of CVD (HR: 0.84; 95% CI: 0.74 - 0.96) (36). In contrast, no association was found between higher adherence to the MIND score and the risk of HTN (HR: 1.08; 95% CI: 0.86 - 1.36) in another prospective cohort study (37).

### 4.6. Nordic Diet Score

One cohort study examined the association between the Nordic score and CVD (38). The Nordic score, developed by Kanerva et al. (47), includes nine components: fruit and berries (apple, pear, strawberry, mulberry, and cranberry), vegetables (tomato, cucumber, cabbage, roots, peas, and leafy vegetables), cereals (oat, rye, barley, and whole grains), low-fat milk, fish, meat products (red meat and processed meat products), alcohol, total fat, and the fat ratio (i.e., the ratio of PUFA to SFA plus trans-fatty acids). The cohort’s findings showed a lower risk of CVD in participants in the third (HR: 0.65; 95% CI: 0.45 - 0.95) and fourth (HR: 0.40; 95% CI: 0.27 - 0.65) quartiles of the Nordic score than those in the first quartile.

### 4.7. Portfolio Diet Score

One study addressed the Portfolio diet score and investigated its link with T2DM (41). The Glenn et al. (48) study was used to compute the Portfolio diet score. They designed this score based on six food components, including plant protein, viscous fiber, nuts, phytosterols, monounsaturated fatty acids, and saturated fats/cholesterol. The above cohort was conducted on 2,188 adults who were followed for 8.9 years. Its results demonstrated a null relationship between the Portfolio diet score and the risk of T2DM in adults (HR: 0.91; 95% CI: 0.64 - 1.31). We found no other published prospective cohort in this issue.

## 5. Discussion

This systematic review synthesized the evidence from the TLGS on the associations between various plant-based dietary scores and cardiometabolic diseases in a large cohort of Iranian adults. The principal finding is that the associations are diet-specific and outcome-dependent. The MIND and Nordic diets demonstrated significant, protective associations with CVD, while the DASH score was related to a lower risk of IR. Conversely, the evidence for the MeDi was largely non-significant, and the Portfolio diet showed no association with T2DM risk.

Among studies investigating the MeDi and cardiometabolic disease derived from TLGS, only one identified a significant inverse association with T2DM in the highest category of MeDi adherence (32). However, a subsequent prospective analysis with a larger sample size and a comparable follow-up duration found no significant association between the MeDi score and T2DM risk (32). This discrepancy may be attributed to key methodological differences, as the earlier study defined nuts as a distinct food group and did not incorporate dairy intake into its scoring (32). Furthermore, the latter study adjusted for a more comprehensive set of potential confounders, such as smoking and physical activity, which can attenuate observed effects (35). In general, TLGS studies do not provide strong evidence that there is a link between the MeDi score and cardiometabolic diseases. The observed null findings in most of the included studies may be partially attributable to the methodological constraint of assessing MeDi adherence using population-specific median consumption, which can limit cross-population comparability. However, the persistence of non-significant results even when applying the Sofi et al. (43) scoring system — which uses absolute cut-off values to enhance discriminatory power — in a study suggests that other factors beyond mere quantitative differences in food intake are likely involved (29). The MeDi represents a holistic lifestyle and dietary pattern rooted in a specific cultural and ecological context (49, 50). Fundamental differences between Mediterranean and non-Mediterranean populations — including practices related to food production, composition, preparation, and preference, particularly concerning vegetables and fruits — may significantly influence the diet’s overall health effects. Quantitative dietary assessments alone may not capture these qualitative and contextual dimensions, which could potentially influence the associations of the MeDi score in non-Mediterranean populations (29, 49).

The DASH diet was originally developed and validated in U.S. populations and is now an established non-pharmacological intervention for HTN management (44). The efficacy of the DASH diet for broader cardiometabolic risk reduction remains uncertain due to inconsistent findings from observational studies, a generally low quality of evidence, and a limited number of investigations (19, 51, 52). The TLGS studies found no significant link between the DASH score and the incidence of HTN or T2DM (34, 35, 37, 39). However, one study did report a significant inverse association between the highest level of DASH adherence and IR (30). This aligns with previous research suggesting that the dietary benefits of DASH in observational studies are only apparent with a very high degree of adherence (53, 54).

Tehran Lipid and Glucose Study studies indicate a protective association between the MIND and Nordic diets and the occurrence of CVD events (36, 38). The MIND diet is a hybrid of the MeDi and DASH diets, specifically designed to support neurocognitive health. It distinctively prioritizes the consumption of berries and green leafy vegetables, while demonstrating less emphasis regarding other food groups — such as total fruit, dairy, potatoes, and fish — that are emphasized more strongly in the MeDi and DASH diets (46).

The Nordic diet is a popular dietary pattern in Scandinavia and other northern European countries. Similar to the MeDi, it advocates a high intake of fruits, vegetables, whole grains, and fatty fish while recommending limited consumption of red and processed meats and saturated fatty acids. However, differences shaped by regional food availability result in distinct phytochemical profiles and fatty acid compositions between the two diets. A primary distinction lies in the principal culinary oils: Olive oil is central to the MeDi, whereas canola (rapeseed) oil is characteristic of the Nordic diet. Therefore, the Nordic diet provides a more balanced ratio of monounsaturated and polyunsaturated fats, including significant omega-3 fatty acids from both rapeseed oil and fatty fish (55).

The Portfolio diet is a therapeutic pattern designed to lower low-density lipoprotein cholesterol by incorporating multiple cholesterol-lowering foods (56). In the TLGS cohort, one study found no significant association between this diet and the incidence of T2DM over 8.9 years of follow-up (41). This null finding may be because the diet's primary mechanisms — such as bile acid binding and reduced cholesterol absorption — more directly target dyslipidemia than pathways of glucose metabolism.

This review is subject to several limitations. The observational design of all included studies prevents causal inference, and residual confounding from unmeasured or imperfectly measured lifestyle factors remains a possibility. Furthermore, dietary indices in most studies were assessed only at baseline, despite the fact that diets may alter over time as a result of changes in socioeconomic factors. Additionally, the calculation of MeDi and MIND scores required modification due to a lack of data on primary culinary oils and alcohol consumption.

### 5.1. Conclusions

With the exception of a single study reporting a lower risk of T2DM with high MeDi adherence, the findings from the TLGS do not support a beneficial association between adherence to the MeDi or DASH diets and the incidence of T2DM or HTN. In contrast, the MIND and Nordic diets demonstrated significant inverse associations with CVDs, indicating their promise for primary prevention in the Iranian adult population. Due to the limited research on obesity, dyslipidemia, and CVD endpoints, the association between plant-based diets and these outcomes necessitates additional examination within the TLGS framework.

## Data Availability

The dataset presented in the study is available on request from the corresponding author during submission or after publication. The data are not publicly available due to privacy or ethical restrictions.
